# Effect of intensive insulin therapy on first-phase insulin secretion in newly diagnosed type 2 diabetic patients with a family history of the disease

**DOI:** 10.3892/etm.2014.2114

**Published:** 2014-12-08

**Authors:** QING LI, LUAN WANG, LIN XIAO, ZHONGCHAO WANG, FANG WANG, XIAOLONG YU, SHENGLI YAN, YANGANG WANG

**Affiliations:** 1 Department of Clinical Laboratory, The First People’s Hospital of Zibo City, Zibo, Shandong 255200, P.R. China; 2 Department of Endocrinology, Stem Cell Research Center, The Affiliated Hospital of Medical College, Qingdao University, Qingdao, Shandong 266003, P.R. China; 3 Biochemistry Office, Weifang Medical College, Weifang, Shandong 261042, P.R. China

**Keywords:** type 2 diabetes, intensive insulin therapy, family history, β-cell function, first-phase insulin secretion, insulin resistance

## Abstract

Intensive insulin treatment is known to improve β-cell function in the majority of patients with newly diagnosed type 2 diabetes mellitus (T2DM), and family history (FH) is known to be an important independent risk factor for T2DM. Thus, the aim of the present study was to investigate the difference in first-phase insulin secretion and the effect of intensive insulin therapy on the improvement of β-cell function between T2DM patients with and without a FH of diabetes. Patients with newly diagnosed T2DM and healthy controls were divided into groups according to their FH of diabetes. Improvement in β-cell function was evaluated with an arginine stimulation test after two weeks of continuous subcutaneous insulin infusion (CSII). Compared with the control group, the level of fasting insulin and the homeostasis model assessment of insulin resistance (HOMA2-IR) were higher in the DM group, while the homeostasis model assessment of β-cell insulin secretion (HOMA2-%β) and the first-phase peak ratio were lower (P<0.05). In addition, the first-phase peak ratio in the FH- control group was higher compared with that in the FH+ control group (P=0.023). Following CSII, all the patients achieved excellent blood glucose control in 6.2±3.6 days, without severe adverse effects. In the DM groups, the fasting insulin level and HOMA2-IR were lower, while the HOMA2-%β and first-phase peak ratio were higher, when compared with the values prior to treatment, particularly in the FH- DM group. The HOMA2-%β in the FH+ DM group was lower compared with the FH- DM group (P=0.027). Therefore, T2DM patients with and without a FH of the disease were shown to have a good response to CSII in the improvement of insulin resistance and β-cell function; however, the improvements were less significant in patients with a FH compared with patients without a FH of diabetes.

## Introduction

The natural course of type 2 diabetes mellitus (T2DM) is longer than that observed in the clinic, and consists of progression from normal glucose tolerance (NGT) to impaired glucose tolerance (IGT), ultimately leading to T2DM. Among individuals with hyperglycemia, early insulin secretion has been shown to decrease by 27% from NGT to IGT, and decrease by an additional 51% from IGT to T2DM (1). Progressive deterioration of pancreatic β-cell function and the worsening of hyperglycemia over time are the basic characteristics of T2DM. Intensive insulin treatment (IIT) can decrease the endogenous secretory demand on β-cells, which may lead to the recovery of β-cell function and possibly prevent further loss of β-cell mass (2,3). A series of studies have confirmed that the early implementation of IIT can markedly improve β-cell function in the majority of patients with newly diagnosed T2DM (4–6). Furthermore, the early recovery of β-cell function and glycemic control through IIT improves unsatisfactory metabolic outcomes and reduces the risk of diabetic complications (7,8). In the Steno-2 study and UK Prospective Diabetes Study, patients exhibiting near-normal glycemic control from the diagnosis of T2DM were reported to have a lower long-term cardiovascular mortality rate compared with patients with worse initial control (9,10). However, the mechanisms responsible for this disease-modifying effect remain unclear.

It is well recognized that impaired first-phase insulin secretion is an early marker of β-cell dysfunction, and also an independent and additive predictor of the progression of diabetes. At present, glucotoxicity is considered to restrict first-phase insulin secretion, leading to decreased second-phase insulin secretion and potentially an increased rate of β-cell apoptosis (11,12). Full or partial recovery of first-phase insulin secretion may aid long-term maintenance of good glycemic control. Weng *et al* previously reported that first-phase insulin secretion was partially restored following the completion of intensive therapy, and the improvement in β-cell function was associated with the persistence of euglycemia for one year (13).

T2DM is a multi-factorial disease associated with several possible risk factors, including life style, increasing age, insulin resistance, family history (FH) of diabetes and ethnicity. FH is known to be an important independent risk factor for T2DM, and is ascribed to shared genes and a shared environment (14,15). The probability of developing T2DM is two to four fold higher for individuals with a positive FH compared with those without, depending on the number of affected family members and their relationship to the patient (16–18). However, the affected degree and exact mechanism are not clear.

In the present prospective study, the differences in first-phase insulin secretion and the effect of ITT on the improvement of β-cell function were investigated and compared in newly diagnosed T2DM patients with or without a FH of diabetes.

## Materials and methods

### Patients

Patients with newly diagnosed T2DM were recruited from outpatient and inpatient clinics of the Department of Endocrinology at the Affiliated Hospital of Medical College, Qingdao University (Qingdao, China), between January 2011 and January 2013. In total, 360 patients were screened for enrollment. Of those patients, 307 patients met the inclusion criteria and were personally interviewed, with 300 patients ultimately enrolled in the study. Patients were divided into two groups according to their FH of diabetes. A total of 95 patients comprised the positive FH group (FH+ DM group), while the remaining 205 patients participated in negative FH group (FH- DM group). A positive FH was defined as a direct or collateral relative with DM within three generations of the patient from the maternal or paternal side. Individuals that had undergone a health examination in our hospital were screened as controls and 256 healthy volunteers were enrolled in the study. All the controls were divided into two groups according to their FH of diabetes. In total, 91 participants were included in the positive FH group (FH+ control group), while the remaining 165 healthy volunteers comprised the negative FH group (FH- control group). All the participants were subsequently enrolled and underwent treatment until March 2013 at the Department of Endocrinology at the Affiliated Hospital of Medical College, Qingdao University. The study protocol was approved by the Ethical Committee of the Affiliated Hospital of Medical College, Qingdao University, and informed consent, according to the Declaration of Helsinki, was provided by every participant.

Male and female patients, aged between 30 and 60 years, were included in the study. All the patients had received a clinical and laboratory diagnosis of T2DM, according to the criteria of the American Diabetes Association (19), and were newly diagnosed without having undergone antidiabetic therapy. Patients with type 1 or other types of diabetes, or T2DM complicated with diabetic nephropathy or diabetic retinopathy, sustained hypertension, unstable angina or stroke, recent myocardial infarction (<6 months), heart failure, peripheral vascular disease, acute or chronic infections, cancer, hepatic or renal disease and mental disorders were excluded from the study. In addition, patients were excluded if pregnant or breast-feeding, or receiving medications affecting glucose and insulin levels.

The control groups comprised male and female patients aged between 30 and 60 years. Each volunteer had been found to have a normal glucose tolerance via an oral glucose tolerance test (OGTT). Volunteers with any types of diabetes, sustained hypertension, unstable angina or stroke, recent myocardial infarction (<6 months), heart failure, peripheral vascular disease, acute or chronic infections, cancer, pregnancy or breast-feeding, hepatic or renal disease, mental disorders or those receiving medications affecting glucose and insulin levels were excluded from the study.

### Treatment procedure

Prior to enrollment, the diabetic and control subjects underwent careful physical examinations and detailed laboratory examinations to exclude any condition that may interfere with glucose tolerance. Subsequently, β-cell function was evaluated in the controls using an arginine stimulation test.

**Figure 1:**
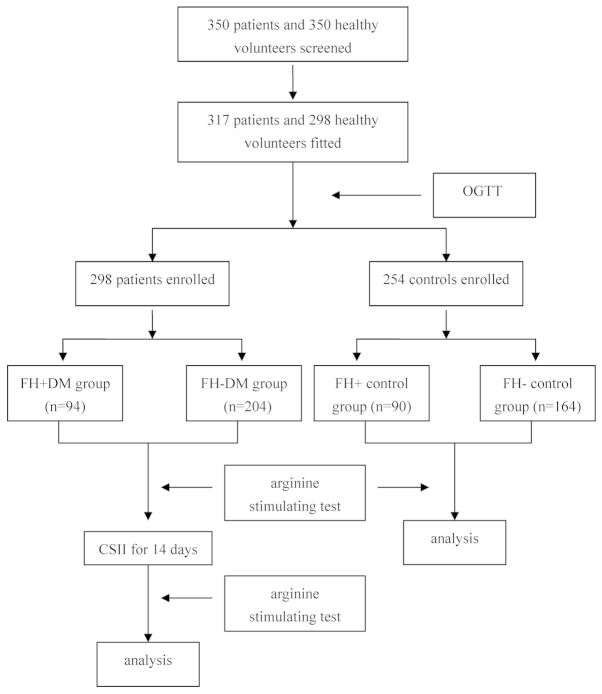
Treatment procedure for the trial. FH, family history; DM, diabetes mellitus; CSII, continuous subcutaneous insulin infusion; OGTT, oral glucose tolerance test.

All the patients were admitted to hospital and recommended a diabetic diet and an exercise routine (walking or similar for 1 h three times per week during the entire study). For two weeks, the patients underwent ITT with continuous subcutaneous insulin infusion (CSII) to reach and maintain an excellent glycemic control, which was defined as a fasting blood glucose level of <5.6 mmol/l and a postprandial blood glucose level of <7.8 mmol/l. At day two after the termination of ITT, and without administration of additional medications that may have affected the glucose and insulin levels, the β-cell function was reassessed (Fig. 1).

### Measurement

Upon enrollment, the medical history, body weight, height, blood pressure, waist circumference, hip circumference, body mass index (BMI) and waist to hip ratio were recorded for each patient. The waist circumferences were measured to the nearest 0.1 cm at the narrowest point between the lowest rib and the uppermost lateral border of the right iliac crest. Blood pressure was measured in the supine position on the right arm three times using a mercury manometer (Mercury Sphygmomanometer SB3001A; Wenzhou Doctor Medical Device Co., Ltd., Wenzhou, China) following a 20-min rest, and the mean of three measurements was used for analysis. The BMI was calculated as the weight divided by the squared height (kg/m^2^).

Levels of fasting plasma glucose (FPG), postprandial plasma glucose (PPG), glycosylated hemoglobin (HbA1c), glutamic acid decarboxylase antibody, free fatty acid (FFA), insulin and C-peptide, as well as the lipid profile and the first-phase insulin secretion, were measured prior to CSII and at day two following insulin cessation with a 10-h overnight fast. The PPG level was measured at 2 h after the main meals in hospital. OGTT was performed according to the World Health Organization standard (20). After 10–12 h of overnight fasting, subjects ingested a solution containing 75 g dextrose over a 5-min period. Venous blood samples were collected at 0, 30, 60 and 120 min for the determination of plasma glucose by an automated glucose oxidase method (Glucose Analyzer 2; Beckman Instruments, Fullerton, CA, USA) according to the manufacturer’s instructions.

First-phase insulin secretion was assessed with an arginine stimulation test at 8:00am, after a 10–12-h overnight fast. A 25% solution of L-arginine (5 g/20 ml; Shanghai Xinyi Jinzhu Pharmaceutical Co., Ltd., Shanghai, China) was infused intravenously in 30 sec. Blood samples for the determination of serum insulin and C-peptide levels were collected prior to initiating the infusion and at 2, 4 and 6 min after the infusion. Serum samples were measured using the Roche Modular system and an electrochemiluminescence immunoassay kit (Roche Diagnostics GmbH, Mannheim, Germany). This assay shows 0.05% cross-reactivity to intact human proinsulin and the primary circulating split form, des 31,32-proinsulin.

### Calculations

Based on the updated homeostasis model assessment (HOMA) methods, the HOMA insulin resistance (HOMA2-IR) and HOMA β-cell insulin secretion (HOMA2-%β) were calculated using HOMA2 calculator version 2.2 software (http://www.dtu.ox.ac.uk/homacalculator/index.php). The estimated first-phase insulin secretion was assessed by the first-phase peak ratio as follows: Peak insulin/fasting insulin.

### Adverse events

Adverse events were documented throughout the study. Weight was assessed using a medical scale (HW600B, Zhengzhou Kaiyuan Electronic Co., Ltd., Zhengzhou, China) to avoid errors. Mild hypoglycemic episodes were defined as symptoms indicative of low blood glucose, accompanied by a documented capillary blood glucose value of ≤70 mg/dl. Severe hypoglycemia was defined as symptoms of hypoglycemia that required assistance from another individual for treatment, regardless of the capillary blood glucose level.

### Statistical analysis

Statistical analysis was performed using SPSS 17.0 software (SPSS, Inc., Chicago, IL, USA). Data are presented as the mean ± standard error of the mean. Parameters that did not fulfill normal distribution were mathematically transformed to improve the symmetry for subsequent analyses. Baseline characteristics of the T2DM and control subjects were compared using the independent sample t-test or χ^2^ test. The differences between variables prior to and following intensive glycemic control in the T2DM subgroup were analyzed for significance using a paired sample t-test. The associations between variables were analyzed by simple correlation (Pearson’s or Spearman’s correlation analysis) and multiple regression in a stepwise forward manner. All the statistical analyses were two-sided and P<0.05 was considered to indicate a statistically significant difference.

## Results

### Subject characteristics

In total, 300 patients with newly diagnosed T2DM and 256 healthy volunteers completed this study. Their baseline data are summarized in Table I. No statistically significant differences were observed with regard to the age, gender, BMI, systolic blood pressure, diastolic blood pressure, and levels of triglyceride, total cholesterol, high-density lipoprotein-cholesterol and low density lipoprotein-cholesterol between the T2DM groups and the respective control groups prior to treatment. However, the levels of HbA1c, blood glucose, FFA and HOMA2-IR in the FH+ DM group and FH- DM group were higher when compared with the respective FH+ and FH- control groups (P<0.05). In addition, the HOMA2-%β in the FH+ DM and FH- DM groups was markedly lower compared with the FH+ and FH- control groups (P<0.05). No statistically significant differences were observed in age, HbA1c, FPG, PPG, HOMA2-%β and HOMA2-IR between the FH+ DM and FH- DM groups. However, the HOMA2-%β was found to be higher in the FH- control group when compared with the FH+ control group (P=0.043).

### First-phase insulin secretion prior to therapy

Fasting insulin levels in the FH+ DM and FH- DM groups were significantly higher compared with the levels in the respective FH+ and FH- control groups (P<0.05), while there was no significant statistical difference observed between the FH+ DM group and FH- DM groups (10.49±6.14 vs. 10.01±6.47; P=0.135). Following an infusion of arginine, insulin secretion reached the highest level at 2 min, after which the insulin levels began to decrease. The first-phase peak ratios in the FH+ DM group, FH- DM group and FH+ and FH- control groups were 5.03, 5.23, 7.29 and 8.88, respectively. The first-phase peak ratios in the FH+ DM and FH- DM groups were significantly lower compared with the FH+ and FH- control groups (P<0.05). Compared with the FH- control group, the first-phase peak ratio in the FH+ control group was statistically lower (P=0.023), while no statistically significant difference was observed between the FH+ DM and FH- DM groups (Table II).

### Effect of CSII on glycemic control

Prior to treatment with CSII, the blood glucose levels were high in the diabetic patients, with an average FPG of 10.48±3.68 mmol/l in the FH+ DM group and 9.94±1.99 mmol/l in the FH- DM group. In addition, the average PPG level was 13.67±4.35 mmol/l in the FH+ DM group and 13.16±6.21 mmol/l in the FH- DM group, while the average level of HbA1c was 8.34±2.10% in the FH+ DM group and 8.51±2.37% in the FH- DM group. Following treatment with CSII, all the patients achieved excellent blood glucose control in 6.2±3.6 days. The FPG and PPG levels were significantly reduced (FGP: FH+ DM group, 10.48±3.68 vs. 5.38±0.6 mmol/l; FH- DM group, 9.94±1.99 vs. 5.56±1.77 mmol/l; PPG: FH+ DM group, 13.67±4.35 vs. 6.89±1.05 mmol/l; FH- DM group, 13.16±6.21 vs. 6.76±0.43 mmol/l; P<0.05), with an average daily insulin dose of 0.8 U/kg (range, 0.32–1.46 U/kg).

### Effect of CSII on insulin resistance and β-cell function

**Figure 2:**
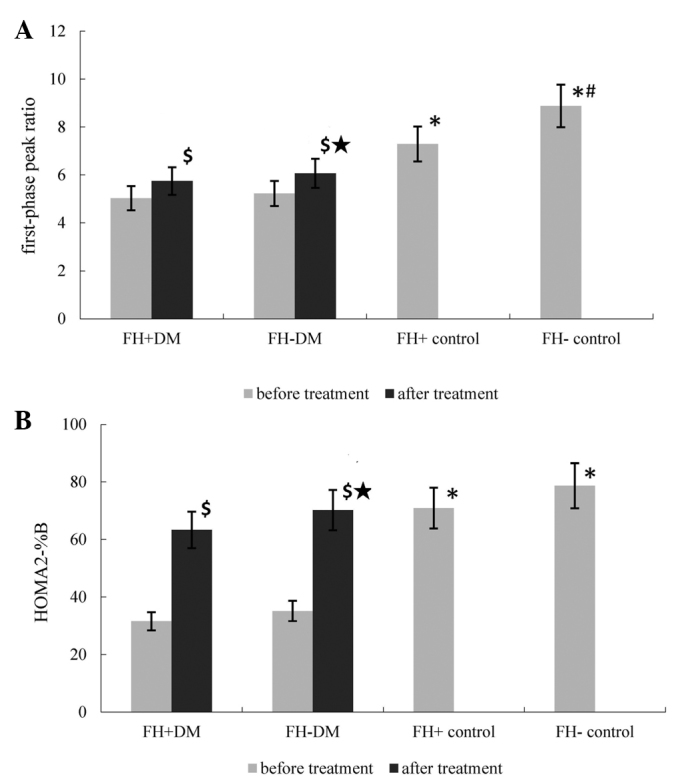
Differences in the (A) first-phase peak ratio and (B) HOMA2-%β among the groups. Prior to treatment, the first-phase peak ratio and HOMA2-%β were markedly lower in the FH+ DM and FH- DM groups when compared with the FH+ and FH- control groups (^*^P<0.05). Compared with the FH- control group, the first-phase peak ratio in the FH+ control group was significantly lower (^#^P=0.023). Following CSII, the first-phase peak ratio and HOMA2-%β were higher in the DM groups compared with the value pretreatment (^$^P=0.037, 0.042, 0.023 and 0.019, vs. pretreatment value for the first-phase peak ratio and HOMA2-%β in the FH+ DM and FH- DM groups, respectively). The first-phase peak ratio and HOMA2-%β were higher in the FH- DM group compared with the FH+ DM group (^«^P=0.044 and 0.027, respectively). FH, family history; DM, diabetes mellitus; HOMA2-%β, homeostasis model assessment of β-cell insulin secretion; CSII, continuous subcutaneous insulin infusion.

**Figure 3:**
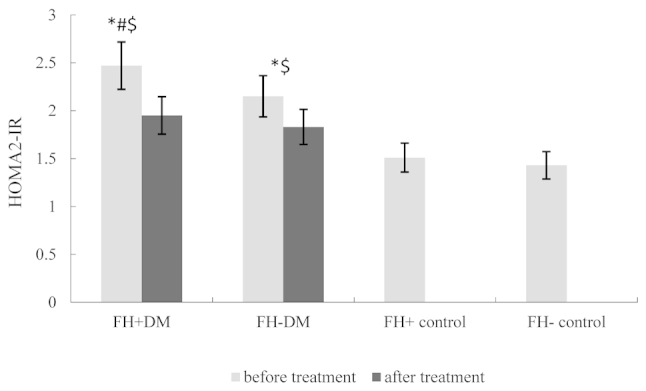
Differences in the HOMA2-IR among the groups. Prior to treatment, the HOMA2-IR in the FH+ DM and FH- DM groups was significantly higher compared with the FH+ and FH- control groups (^*^P<0.05). The HOMA2-IR was higher in the FH+ DM group compared with the FH- DM group (^#^P=0.032). Following CSII, the HOMA2-IR in the FH+ DM and FH- DM groups was lower compared with the value pretreatment (^$^P=0.024 and 0.019, respectively); however, no statistically significant difference was observed between the two DM groups (P=0.327). FH, family history; DM, diabetes mellitus; HOMA2-IR, homeostasis model assessment of insulin resistance; CSII, continuous subcutaneous insulin infusion.

At day two following the end of therapy, the fasting insulin levels of the patients in the FH+ DM group and FH- DM group were lower compared with the value prior to therapy (FH+ DM group, 8.69±3.22 vs. 10.49±6.14 mIU/l; FH- DM group, 8.46±3.55 vs. 10.01±6.47 mIU/l; P=0.013 and 0.022, respectively), while the first-phase peak ratios in the two groups were higher than the value prior to treatment (FH+ DM group, 5.75±2.04 vs. 5.03±2.51; FH- DM group, 6.17±2.42 vs. 5.23±2.47; P=0.037 and 0.042, respectively). The first-phase peak ratio in the FH- DM group was higher compared with the FH+ DM group (P=0.049), as shown in Table III and Fig. 2. The HOMA2-IR in the FH+ DM group and FH- DM group was lower compared with the value prior to CSII (FH+ DM group, 1.95±0.62 vs. 2.47±1.09; FH- DM group, 1.83±0.45 vs. 2.15±1.06; P=0.024 and 0.019, respectively); however, no statistically significant difference was observed between the two diabetic groups (Fig. 3). The HOMA2-%β in the FH+ DM and FH- DM groups was higher compared with the value prior to therapy (FH+ DM group, 63.37±17.25 vs. 35.15±9.68; FH- DM group, 70.23±19.7 vs. 34.58±7.92; P=0.023 and 0.019, respectively). The HOMA2-%β in the FH+ DM group was lower compared with the FH- DM group (P=0.027; Fig. 2).

### Adverse events

No severe adverse events occurred during the study period. Mild symptoms of hypoglycemia were observed in 27 patients; however, following ingestion of a 20-g cracker, the symptoms were relieved.

## Discussion

A FH of diabetes is not only a risk factor for the disease, but is also positively associated with risk awareness. Individuals with or without a FH of T2DM have been shown to have different pathophysiological characteristics during disease progression (15). In immediate relatives of individuals with T2DM, insulin resistance has been shown to already exist when the glucose levels are normal, and dysfunction in insulin secretion has been found to be a key factor in determining the progression of glucose intolerance (21,22). In the present study, the HOMA2-IR in the FH+ control group was comparable with the FH- group, while the HOMA2-%β and first-phase peak ratio were lower compared with the FH- control group, indicating that immediate relatives of individuals with T2DM already exhibit impaired β-cell function despite being euglycemic.

The progressive deterioration of insulin secretory function in individuals with T2DM is accompanied by a loss of β-cell mass. However, the precise pathological mechanisms leading to β-cell failure are yet to be fully elucidated. A number of factors, including glucotoxicity, lipotoxicity, islet inflammation and amyloid deposition, have been implicated as potentially contributing to this process. The strategy of administering a short course of IIT has been studied in patients with newly diagnosed T2DM (23–25). These studies demonstrated that short-term IIT, delivered by multiple daily injections or CSII, can significantly improve β-cell function in the majority of newly diagnosed patients. In the present study, the therapeutic effect was further confirmed on newly diagnosed T2DM patients with and without a FH of T2DM.

The mechanism by which ITT may improve β-cell function remains unclear. The elimination of glucotoxicity may not be the sole basis for this improvement, and other properties of insulin, including its antilipolytic, anti-inflammatory and antiapoptotic effects, may also contribute to the improved β-cell function (6,8). Li *et al* used a rat model of diabetes, induced by streptozotocin and high-fat feeding, to investigate the protective role of insulin on β-cell function. The authors found that insulin therapy was able to improve β-cell function, markedly reduce the islet fat content and increase the β-cell area through decreasing the rate of apoptosis and increasing the rate of β-cell proliferation (26).

In the present study, all the patients achieved good glycemic control within a mean duration of six days following CSII. The fasting insulin levels, first-phase peak ratio, HOMA2-IR and HOMA2-%β in the patients were all markedly improved compared with the values prior to therapy, which indicated that insulin resistance and β-cell function had been improved. Weng *et al* previously demonstrated that improvements in β-cell function were associated with the persistence of euglycemia for one year, and suggested that the preservation of first-phase insulin secretion is likely to contribute to the higher rates of remission achieved with ITT (13). However, further study is required to confirm this hypothesis.

In the present study, following treatment with CSII and delamination by FH, the HOMA2-%β and first-phase peak ratio were found to be markedly higher than the levels prior to therapy, but remained lower compared with the FH- DM group. In addition, the HOMA2-IR in the FH+ DM group was markedly lower compared with the pretreatment value and comparative with the FH- DM group. These results indicate that defects in β-cell secretion and insulin sensitivity in T2DM patients with a FH of the disease were more severe compared with T2DM patients without a FH.

A limitation of the current study was the absence of a long-term follow-up period; thus, the durability of the beneficial effect of short-term CSII on β-cell function and glycemic control in T2DM patients remains to be defined. An additional limitation was the use of surrogate indices (e.g. euglycemic hyperinsulinemic glucose clamp) for the assessment of insulin secretion and insulin sensitivity.

In conclusion, the present study investigated the differences in response to ITT between T2DM and healthy controls with or without a FH of diabetes. T2DM patients, irrespective of their FH, were found to have a good response to CSII via the improvement of insulin resistance and β-cell function. However, the improvements observed in patients with a FH of diabetes were less significant compared with the T2DM patients without a FH. In addition, for the healthy individuals included in the study, a FH of T2DM was shown to have an important effect on disease progression.

## Figures and Tables

**Table I:** Patient baseline characteristics in the four groups.

Characteristics	FH+ DM group	FH- DM group	FH+ control group	FH- control group
Male/female (n)	61/34	135/70	56/35	103/62
Age (years)	46.04±8.63	46.63±7.87	47.44±5.65	46.40±6.31
BMI (kg/m^2^)	27.04±5.80	26.80±3.53	26.76±3.65	27.30±2.39
SBP (mmHg)	131±15	129±15	128±15	130±19
DBP (mmHg)	86±10	84±10	84±11	85±11
TG (mmol/l)	2.84±4.33	2.73±2.98	2.75±0.36	2.82±0.46
TC (mmol/l)	5.31±1.52	5.20±2.23	5.33±0.50	5.04±0.92
HDL-c (mmol/l)	1.25±0.03	1.21±0.37	1.29±0.34	1.32±0.14
LDL-c (mmol/l)	3.64±1.17	3.67±1.23	3.57±0.56	3.54±0.78
HbA1c (%)	8.34±2.10^a^	8.51±2.37^a^	5.21±1.19	5.39±1.31
FPG (mmol/l)	10.48±3.68^a^	9.94±1.99^a^	5.22±0.46	5.15±0.50
PPG (mmol/l)	13.67±4.35^a^	13.16±6.21^a^	6.35±1.56	6.84±1.24
FFA (mmol/l)	0.84±0.68^a^	0.87±0.96^a^	0.57±0.17	0.58±0.24
HOMA2-IR	2.47±1.09^a^	2.35±1.06^a^	1.51±0.66	1.43±0.42
HOMA2-%β	34.58±7.92^a^	35.15±9.68^a^	70.91±15.3	78.67±16.84^b^

a P<0.05, vs. respective control group.

No statistically significant differences were identified between the FH- DM and FH+ DM groups.

b P=0.043, vs. FH+ control group.

FH, family history; DM, diabetes mellitus; BMI, body mass index; SBP, systolic blood pressure; DBP, diastolic blood pressure; TG, tryglyceride; TC, total cholesterol; HDL, high-density lipoprotein-cholesterol; LDL, low-density lipoprotein-cholesterol; HbA1c, glycosylated hemoglobin; FPG, fasting plasma glucose; PPG, postprandial plasma glucose; FFA, free fatty acid; HOMA2-IR, homeostasis model assessment of insulin resistance; HOMA2-%β, homeostasis model assessment of β-cell insulin secretion.

**Table II:** First-phase peak ratio in the four groups following L-arginine infusion.

Parameter	FH+ DM group	FH- DM group	FH+ control group	FH- control group
Insulin (mIU/l)				
0 min	10.49±6.14^a^	10.01±6.47^a^	5.83±2.57	5.41±1.83
2 min	49.11±29.35	53.10±29.99	47.64±16.60	47.89±18.54
4 min	35.42±21.61	39.33±20.72	33.95±12.59	36.70±16.59
6 min	25.98±14.61	23.02±13.49	27.57±9.42	27.26±14.01
Peak ratio	5.03±2.51	5.23±2.47	7.29±3.79^a^	8.88±3.32^a^,^b^

a P<0.05, vs. respective control groups;

b P=0.023, vs. FH+ control group.

No statistically significant differences were observed between the FH+ DM group and FH- DM group. FH, family history; DM, diabetes mellitus.

**Table III:** Clinical characteristics of the patients in the DM groups following CSII.

Parameter	FH+ DM group	FH- DM group
FPG (mmol/l)	5.38±0.6	5.56±1.77
PPG (mmol/l)	6.89±1.05	6.76±0.43
Insulin (mIU/l)		
0 min	8.69±3.22	8.46±3.55
2 min	40.38±26.45	53.49±30.87
4 min	26.48±35.53	24.05±10.26
6 min	19.75±17.66	15.70±6.62
Peak ratio	5.75±2.04	6.17±2.42^a^
HOMA2-IR	1.95±0.62	1.83±0.45
HOMA2-%β	63.37±17.25	70.23±19.7^b^

a P=0.049 and

b P=0.027, vs. FH+ DM group.

FH, family history; DM, diabetes mellitus; FPG, fasting plasma glucose; PPG, postprandial plasma glucose; HOMA2-IR, homeostasis model assessment of insulin resistance; HOMA2-%β, homeostasis model assessment of β-cell insulin secretion; CSII, continuous subcutaneous insulin infusion.
